# The combination of *Astragalus membranaceus* and ligustrazine mitigates cerebral ischemia‐reperfusion injury via regulating NR2B‐ERK/CREB signaling

**DOI:** 10.1002/brb3.2867

**Published:** 2022-12-31

**Authors:** Xialing Tang, Shanshan Xie, Huajun Wang, Yingbin Li, Zhiyu Lai, Shuangxi Sun, Ruanhuan Pan, Yan Huang, Jun Cai

**Affiliations:** ^1^ The Second Institute of Clinical Medicine Guangzhou University of Chinese Medicine Guangzhou China; ^2^ Diagnosis and Treatment Center of Encephalopathy Hubei Provincial Hospital of Chinese Medicine Wuhan China; ^3^ Diagnosis and Treatment Center of Encephalopathy Guangdong Provincial Hospital of Chinese Medicine Guangzhou China; ^4^ Department of Neurosurgery, Hospital of Guangzhou University Mega Center Guangdong Provincial Hospital of Chinese Medicine Guangzhou China

**Keywords:** *Astragalus membranaceus*, cerebral ischemia‐reperfusion injury, ligustrazine, N‐methyl‐D‐aspartate receptors, stroke

## Abstract

**Background and purpose:**

Cerebral ischemia‐reperfusion (I/R) injury is a major factor underlying the high mortality and morbidity rates in stroke patients. Our previous study found that the combination of *Astragalus membranaceus* extract and ligustrazine (Ast+Lig) treatment could protect brain tissues against inflammation in rats with thrombolytic cerebral ischemia. Activation of N‐methyl‐D‐aspartate receptors (NMDAR) is implicated in brain damage induced by cerebral I/R injury.

**Methods:**

We used in vivo and in vitro models of cerebral I/R injury for middle cerebral artery occlusion/reperfusion in mice and oxygen‐glucose deprivation/reoxygenation in primary rat cerebral cortical neurons to evaluate the protective effects of Ast+Lig on cerebral I/R injury, and whether the protective mechanism was related to the regulation of NMDAR‐ERK/CREB signaling.

**Results:**

Treatment with Ast+Lig, or MK‐801 (an inhibitor of NMDAR) significantly ameliorated neurological deficits, decreased infarct volumes, suppressed neuronal damage and Ca^2+^ influx, and maintained the mitochondrial membrane potential in vivo and in vitro following cerebral I/R injury based on 2,3,5‐triphenyl tetrazolium chloride staining, immunohistochemistry, and immunofluorescent staining. Furthermore, treatment with Ast+Lig evidently prevented the upregulation of NR2B, but not NR2A, in vivo and in vitro following cerebral I/R injury based on western blotting and reverse transcription‐quantitative PCR analyses. Moreover, treatment with Ast+Lig significantly increased the phosphorylation of ERK and CREB, as well as increasing their mRNA expression levels in vivo and in vitro following cerebral I/R injury.

**Conclusions:**

The overall results thus suggest that the Ast+Lig combination conferred neuroprotective properties against cerebral I/R injury via regulation of the NR2B‐ERK/CREB signaling pathway.

## 1 INTRODUCTION

Ischemic stroke is one of the leading causes of death and permanent disability in adults worldwide, posing a severe threat to public health (Kurnianto et al., 2021). Until recently, the most effective therapeutic modality to decrease mortality and long‐time morbidity in stroke patients was to restore cerebral blood flow in a timely manner (An et al., 2016). Recombinant tissue plasminogen activator (rt‐PA), as an intravenous thrombolytic agent rapidly administered to patients within 4.5 h of ischemic stroke onset remains the mainstay of early treatment of acute ischemic stroke (Gauberti et al., 2021). Since 2015, after the results of the five positive trials (MR CLEAN, EXTEND‐IA, ESCAPE, SWIFT PRIME, and REVASCAT) were published, mechanical thrombectomy became a standard treatment for large vessel occlusion in acute ischemia stroke cases (Palaniswami & Yan, 2015). Even though the development of novel therapeutic modalities to restore cerebral circulation has improved the outcomes of acute cerebral ischemia treatment, many cerebral stroke patients still suffer severe disability (Zelenak et al., 2021).

Cerebral ischemia‐reperfusion (I/R) injury is a pathophysiological cascade that can lead to brain injury and tissue deterioration, which compromises the beneficial effect of recanalization (Costa et al., 2021). Ischemic tissue reperfusion can cause increased reactive oxygen species (ROS), inflammation, necrosis and apoptosis, which aggravate the injury and worsen the prognosis of patients (Guan et al., 2019). In addition, ischemia‐reperfusion can lead to brain edema, brain injury hemorrhage, blood‐brain barrier destruction and neuronal death, aggravating tissue damage (Wang et al., 2019). During cerebral I/R, large amounts of glutamate are released into the brain. Then excess extracellular glutamate results in overinflux of Ca^2+^ and excitotoxicity through overactivation of N‐methyl‐D‐aspartate receptors (NMDARs) (Deutsch et al., 2021; Yang et al., 2021). Generally, NMDAR, an important receptor‐operated Ca^2+^ channel (Lynch & Guttmann, 2001) are complexes consisting of a homodimer of the NR1 subunit and a homodimer of the NR2 or NR3 subunits or a heterodimer of the NR2 and NR3 subunits (Chakraborty et al., 2017; Chen et al., 2017). NR1 is a functional subunit, which constitutes eight different splicing variants depending on the splicing site, and is a basic subunit necessary to ensure the physiological function of NMDAR ion channels (Kvist et al., 2013). NR2B receptor is usually located in the outer synaptic site, which is mainly related to synaptic inhibition and apoptosis (Maqsood & Stone, 2016). Neuronal apoptosis induced by NMDA excitotoxicity is mainly related to NR2B subunit (Vieira et al., 2016). During cerebral I/R injury, NR2B, primarily distributed in extrasynaptic NMDAR sites, is overactivated and then suppresses Ras‐extracellular signal‐regulated kinase (ERK) signaling through stimulating calmodulin‐dependent protein kinase II, eventually resulting in neuron death (Bustos et al., 2017; El Gaamouch et al., 2012; Kaur et al., 2020). Other studies have also found that hippocampal neuronal apoptosis injury can be alleviated by inhibiting the NR2B receptor and activating the phosphatidylinositol 3‐kinase(PI3K)/protein kinase B(AKT)/mammalian target of the rapamycin (mTOR) signaling pathway (Wang et al., 2014). Moreover, NMDA induces the destruction of tight junction proteins in mouse cerebral vascular endothelial cells through modulation of the MEK/ERK1/2 signal pathway (Mao et al., 2022). In addition, the neuroprotective effect of glutamate on hippocampal neurons is related to the phosphorylation of CREB by calmodulin‐dependent protein kinase (Valera et al., 2008). Numerous studies have shown that the ERK signaling pathway is essential for NMDAR‐dependent neuronal plasticity and survival through regulating CREB activation (Li et al., 2014; Liu et al., 2017; Wu et al., 2020) and NR2B subunits are related to the activation and inhibition of ERK (Sava et al., 2012). Therefore, the ERK/CREB pathway was selected for analysis in the present study. Thus far, neuroprotective drugs against extrasynaptic NMDARs such as a nonselective NMDAR blocker (MK‐801) and NR2B subtype‐specific antagonists ifenprodil and Ro 256981 have been used clinically (Chen et al., 2008; Esposito et al., 2020; Hardingham & Bading, 2010). However, the clinical side effects, such as headaches, heart palpitations, and loss of appetite, significantly limit their clinical use (Smith et al., 2016). Thus, safe and effective neuroprotective drugs for cerebral I/R injury are urgently required.

*Astragalus membranaceus* (Ast) and ligustrazine (Lig), widely used Chinese medicines, are known to possess anti‐inflammatory, antioxidant, and antiapoptotic effects (Khan et al., 2021; Ma et al., 2020; Wang et al., 2020). Our previous study showed that the combination of Ast and Lig (Ast+Lig) ameliorates intracranial microhemorrhage by maintaining blood‐brain barrier integrity in cerebrally ischemic rats (Cai et al., 2014). Recently, we also showed that Ast+Lig suppresses the inflammatory response in rats with thrombolytic cerebral ischemia (Pan et al., 2019). In addition, other studies have also concluded that traditional Chinese medicines that can alleviate cerebral I/R injury usually contain ingredients that can tonify Qi and/or activate blood, and Ast+Lig belongs to this family of ingredients (Han et al., 2017). It has been shown that Lig can potentially protect against cerebral I/R injury in rats through the activation of the PI3K/Akt pathway (Ding et al., 2019) and plays a neuroprotective role in oxygen‐glucose deprivation (OGD)‐induced brain microvascular endothelial cell injury (Yang et al., 2017). Several studies have reported that Ast significantly attenuated I/R injury through a variety of mechanisms, including its anti‐inflammatory, antiapoptotic, and antioxidant effects (Li et al., 2019; Yin et al., 2020). However, whether the protective role of Ast+Lig on cerebral I/R injury is associated with regulating NR2B signaling remains unclear.

In the present study, in vivo and in vitro models of cerebral (I/R) injury for middle cerebral artery occlusion/reperfusion (MCAO/R) in mice and OGD/reoxygenation (OGD/R) in primary rat cerebral cortical neurons were used. The aim of this study was to evaluate the protective effects of Ast+Lig on cerebral I/R injury and whether the protective mechanisms were related to the regulation of NR2B signaling.

## 2 . MATERIAL AND METHODS

### 2.1 . Animals and MCAO surgery

Male C57BL/6 mice (7–8 weeks old), weighing 20–25 g, were obtained from the Medical Laboratory Animal Center of Guangdong. All animal experiments and procedures were approved by the Institutional Animal Care and Use Committee of the Guangdong Provincial Hospital of Chinese Medicine (Animal Protocol Approval no. 2019052). The mice were housed in the animal room at 22–24°C with a 12‐h light/dark circle and free access to food and water. MCAO/R surgery was performed as previously described (Wang et al., 2020). Briefly, mice were anesthetized by inhaling 4% isoflurane for induction of anesthesia and this was maintained with 1.5% isoflurane in an air and oxygen mixture with a small animal anesthesia machine (Ruiwode, R540IP). Then, the focal cerebral ischemia was produced by intraluminal occlusion of the right MCA using a silicone‐coated nylon (6.0) monofilament (Doccol Corporation). After 1 h, the occluding filament was withdrawn to allow blood reperfusion. Mice in the Sham group were treated identically, except the MCAs were not occluded. During the operation, the mice were kept at a constant temperature heating pad at 37°C, and the ambient temperature was maintained at 27 ± 0.5°C. Mice were euthanized by injection of pentobarbital sodium (150 mg/kg). Death was confirmed by lack of respiration, heartbeat, and blinking reflex for 2–3 min.

### 2.2 . Experimental design

The mice were randomly allocated into Sham (*n* = 20), MCAO‐1 h/reperfusion 48 h (MCAO/R‐48 h + Normal Saline [NS], *n* = 20), MCAO/R‐48 h + Ast + Lig (*n* = 20), and MCAO/R‐48 h + MK801 (*n* = 20) groups. The Ast for injection (dried *A. membranaceus* (Fisch) Bunge roots) was obtained from Chiatai Qingchunbao Pharmaceutical Company (Batch no. 1112043), and the Lig for injection was obtained from Zhengzhou Cheuk‐Fung Pharmaceutical Company (Batch no. 11101312).

The Ast (10 mg/ml) and Lig injection (the tetramethylpyrazine content was 10 mg/ml) dosages were 2 and 1 ml/kg, respectively. After 1 h of occlusion, saline (0.09 ml, sham group and MCAO group), Ast + Lig group (0.06 ml + 0.03 ml) combination, or MK801 (a nonselective NMDA blocker, 1 mg/kg [0.06 ml], Sigma‐Aldrich; Merck KGaA; cat. no. M107) were injected intraperitoneally. At 48 h after reperfusion, mice from all groups underwent neurological deficit assessments and were euthanized by decapitation.

### 2.3 . Neurobehavioral assessments

As previously described, a five‐point system was used to assess the neurological deficits of the rats at 48 h after MCAO (Bederson et al., 1986; Pan et al., 2017): 0, no neurological deficits; 1, failure to fully extend right forepaw; 2, circling to the opposite side; 3, falling to contralateral side; 4, not able to walk independently; and 5, died.

### 2.4 . 2,3,5‐Triphenyl tetrazolium chloride (TTC) staining

Brains were rapidly removed, and 1 mm thick coronal sections of the entire brain were obtained and stained with 2% TTC at room temperature for 30 min (Amresco Inc.) to evaluate the infarct volume using ImageJ (National Institutes of Health), as described previously (Pan et al., 2017). The infarct volume ratio was calculated as: ([contralateral hemisphere volume – noninfarcted ipsilateral hemisphere volume]/contralateral hemisphere volume) × 100%.

### 2.5 Cerebral water content

All mice were sacrificed after neurobehavioral scoring. The brain tissue was weighed to record the wet weight (WW) and then dried in an oven at 100°C and weighed again to record the dry weight (DW). Cerebral water content was calculated using the following formula: Cerebral water content  =  (WW – DW)/WW × 100%.

### 2.6 Western blotting

Tissue and cell samples were lysed using NP40 lysis buffer (cat. no. P0013F, Beyotime Institute of Biotechnology) and then subjected to 10% SDS‐PAGE and immunoblotting as previously described (Zhang et al., 2019). The membranes were then blocked with 5% nonfat dry milk at 37°C for 1 h. Next, the membrane was incubated overnight at 4°C with primary antibodies against Foxo3a (cat. no. ab47285, Abcam), cleaved‐caspase‐3 (c‐caspase 3; cat. no. ab179517, Abcam), NR2A (cat. no. ab227233, Abcam), NR2B (cat. no. ab254356, Abcam), Bax (cat. no. ab32503, Abcam), Bcl‐2 (cat. no. ab194583, Abcam), CREB (cat. no. ab178322, Abcam), p‐CREB (S133; cat. no. ab32096, Abcam), ERK1/2 (cat. no. ab184699, Abcam), p‐ERK1/2 (cat. no. ab201015, Abcam), and β‐actin (cat. no. ab6276, Abcam) and α‐tubulin (ab7750, Abcam). After incubation with horseradish peroxidase‐labeled secondary antibodies (NO.32260, Goat anti‐Rabbit IgG [H+L] Poly‐HRP Secondary Antibody, HRP, Thermo Fisher Scientific, Inc.), signals were visualized using a chemiluminescence reagent. α‐Tubulin was used as a loading control to normalize protein expression.

### 2.7 Reverse transcription‐quantitative (RT‐qPCR)

After treatment, total cellular or total tissue RNA was extracted using TRIzol^®^ (Invitrogen; Thermo Fisher Scientific, Inc.) according to the manufacturer's protocol. cDNA was synthesized using M‐MLV Reverse Transcriptase according to the manufacturer's protocol (Takara Bio, Inc.). PCR was performed using specific primers with AceQ qPCR SYBR Green MasterMix (Vazyme Biotech Co., Ltd.). The sequences of the primers used were: NR2A forward, 5′‐ACGTGACAGAACGCGAACTT‐3′ and reverse, 5′‐TCAGTGCGGTTCATCAATAACG‐3′; NR2B forward, 5′‐GCCATGAACGAGACTGACCC‐3′ and reverse, 5′‐GCTTCCTGGTCCGTGTCATC‐3′; Bax forward, 5′‐TGAAGACAGGGGCCTTTTTG‐3′ and reverse, 5′‐AATTCGCCGGAGACACTCG‐3′; Bcl‐2 forward, 5′‐ACGTGGACCTCATGGAGTG‐3′ and reverse, 5′‐TGTGTATAGCAATCCCAGGCA‐3′; and GAPDH forward, 5′‐TGGTATCGTGGAAGGACTC‐3′ and reverse, 5′‐AGTAGAGGCAGGGATGATG‐3′. GAPDH was used as the internal control for normalizing mRNA expression. Relative expression was calculated using the 2^−ΔΔCq^ method.

### 2.8 Immunohistochemistry

Immunohistochemistry was performed as previously described (Esposito et al., 2020). Coronal brain sections (5 μm thick) were randomly selected between 0 and 1 mm posterior to the bregma. After deparaffinization and rehydration, the sections were incubated for 15 min at 95°C in 0.01 M citrate buffer (pH 6.0) for antigen retrieval. Following incubation in 0.3% H_2_O_2_ at room temperature for 30 min followed by 5% BSA to quench endogenous peroxidases and prevent nonspecific immunoreactions respectively, the sections were stained overnight at 4°C using a NeuN antibody (cat. no. ab104224, Abcam, diluted with 5%BSA to 1:300). Then the sections were washed with 0.01 M PBS and later incubated with the corresponding secondary antibody (cat. no. 8125, SignalStain® Boost IHC Detection Reagent [HRP, Mouse], Cell Signaling Technology, Inc.) for 1 h at room temperature; the immunoreactivity was visualized by treatment with a Dako Envision kit HRP (cat. no. K4006; Dako; Agilent Technologies, Inc.). Finally, counterstaining was performed using hematoxylin at room temperature for 2–5 min, after which the tissues were dehydrated and mounted. A total of five fields of view were randomly selected under a light microscope, and the percentage of cells positive for the neuron‐specific protein was analyzed using ImageJ, and the average value was calculated.

### 2.9 Primary rat cerebral cortical neuron culture

Primary rat cerebral cortical neurons were isolated from a timed‐pregnancy embryonic day 14–16 Sprague Dawley rat pups, as previously described (Costa et al., 2021). Briefly, pregnant rats were anesthetized with 3% isoflurane and then pups were taken out of the embryo and euthanized by excessive injection of pentobarbital sodium (150 mg/kg). Death was confirmed as above. In addition, the cortices were chopped into small pieces, digested with trypsin‐EDTA (0.125%) for 15 min at 37°C, and then dissociated by repeated passages. Approximately 2 × 10^5^ cells/cm^2^ were seeded into poly‐l‐lysine (10 mg/ml)‐coated plates. Cells were cultured in Neurobasal Medium supplemented with 3% B27 (Invitrogen; Thermo Fisher Scientific, Inc.), 10 U/ml penicillin, 10 U/ml streptomycin, and 0.5 mmol/l glutamine at 37°C in a humidified incubator supplied with 5% CO_2_. Cultures were maintained for 8 days before treatment, and half of the medium was changed every 3 days.

### 2.10 OGD/R treatment

Briefly, primary rat cerebral cortical neurons were washed twice with PBS (pH = 7.4) and then incubated in serum and glucose‐free DMEM (Gibco; Thermo Fisher Scientific, Inc.). Cell cultures were transferred to a hypoxia chamber (containing a gas mixture of 5% CO_2_ and 95% N_2_) at 37°C to form the OGD conditions in cultured neurons. After OGD exposure for 4 h, the cells were subjected to reoxygenation with DMEM containing serum and glucose under normoxic conditions for 24 h. Control cells were cultured in DMEM under normoxic conditions.

### 2.11 Cell viability assay

Cells cultured in 96‐well plates were exposed to 3 h OGD and 24 h reoxygenation. Cells were treated with Ast (0.2, 0.4, or 0.8 mg/ml), Lig (80, 120, or 160 μmol/l), or Mk801 (5, 10, or 15 μmol/l) for 24 h while being reoxygenated. Control cells were cultured in DMEM under normoxic conditions. Cell viability was measured using a Cell Counting Kit‐8 (Beyotime Institute of Biotechnology), according to the manufacturer's instructions.

### 2.12 Measurement of intracellular Ca^2+^ concentrations

The intracellular Ca^2+^ concentration was measured using the Fluo‐3 AM assay (Beyotime Institute of Biotechnology). According to the manufacturer's instructions, treated neurons were stained with 2 μM Fluo‐3 AM at 37°C for 30 min. Then, cells were washed thrice with PBS, resuspended, and analyzed by flow cytometry (NovoExpress,1.5.0, FACSCalibur BD Biosciences, Inc.).

### 2.13 Measurement of mitochondrial membrane potential (MMP)

The MMP was determined using a JC‐1 assay kit (Beyotime Institute of Biotechnology). Under physiological conditions, cells have a high MMP and JC‐1 accumulates in the mitochondrial matrix to form red fluorescent J‐aggregates. Whereas when cells undergo early apoptosis, the mitochondrial membrane potential is lost, which prevents JC‐1 accumulation in the mitochondria, and thus the dye is dispersed throughout the entire cell leading to a shift from red (JC‐1 aggregates) to green fluorescence (JC‐1 monomers). According to the manufacturer's instructions, treated neurons were harvested, washed in ice‐cold PBS, and stained with 2.5 μM JC‐1 for 30 min at 37°C, and the changes in MMP were analyzed by flow cytometry.

### 2.14 Immunofluorescence

Immunofluorescence was performed as previously described (Xie et al., 2018). Briefly, cells were fixed with 4% paraformaldehyde at 4°C for 20 min, permeabilized with 0.5% Triton X‐100 in PBS, and incubated at 4°C with antibodies against NeuN (cat. no. ab104224, Abcam, diluted with 5%BSA to 1:250) and MAP2 (cat. no. ab183830, Abcam, diluted with 5% BSA to 1:250) overnight. Cells were then incubated with Goat anti‐Rabbit IgG (H+L) Secondary Antibody (FITC; cat. no. 31635, diluted with 5%BSA to 1:250) and Goat anti‐Rabbit IgG (H+L) Cross‐Adsorbed Secondary Antibody (Alexa Fluor™ 546; cat. no. A‐11010, diluted with 5% BSA to 1:250, Thermo Fisher Scientific, Inc.) at 37°C for 1 h. Nuclei were stained with DAPI for 5 min and viewed using an LSM780 laser scanning confocal microscope (scale bar = 50 μm)(Carl Zeiss GmbH).

To detect early apoptotic cells, treated neurons were stained with Annexin V‐FITC (Beyotime Institute of Biotechnology) in PBS for 15 min at 37°C, washed with PBS, and incubated with Hoechst 33342 (Beyotime Institute of Biotechnology) to stain the nuclei for 5 min (room temperature ), washed with PBS, and viewed using an LSM780 laser scanning confocal microscope (scale bar = 50 μm).

### 2.15 Statistical analysis

The data were statistically analyzed using SPSS version 25.0 software (IBM Corp.), and graphs were plotted using GraphPad Prism version 10.0 (GraphPad Software, Inc). All measurement data are presented as the means ± standard deviation. A one‐way ANOVA followed by a Bonferroni's post hoc test was used to compare differences among multiple groups if data were normally distributed and normal variance of homogeneity, or a Dunnett's T3 post hoc test was used if the data were normally distributed but the variance of heterogeneity was not normal.

## 3 RESULTS

### 3.1 Ast+Lig treatment ameliorates neurological deficits and decreases infarct volumes following cerebral I/R injury

**FIGURE 1:**
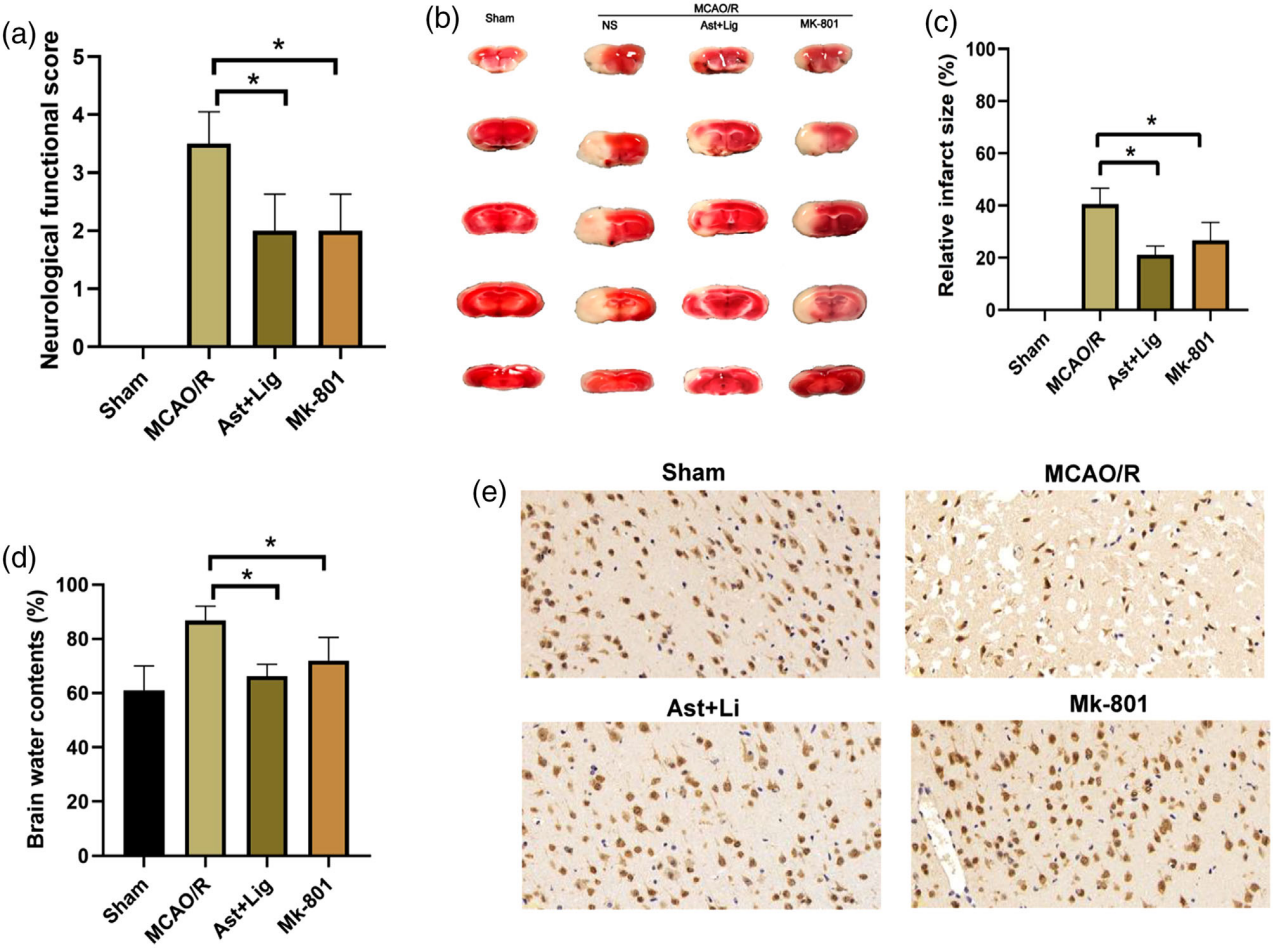
Ast+Lig treatment ameliorated the neurological deficit and decreased the infarct volumes following cerebral I/R injury. (a) Neurological scores were assessed at 48 h after MCAO/R (*n* = 8 per group, *F* = 45.000). (b) Representative TTC‐stained coronal sections of mice brains 48 h after MCAO/R. Pale areas represent infarcted areas. (c) Quantification of infarct volume 48 h after MCAO/R (*n* = 8 per group, *F* = 172.509). (d) Determination of cerebral water content (*n* = 8 per group, *F* = 9.794). (e) Immunohistochemical staining for NeuN in the peri‐infarcted area 48 h after MCAO/R. (f) Quantification of NeuN mean density in each group (*n* = 3 per group, *F* = 403.319). ^*^ *p* < .05 vs. MCAO/R group. Ast, *Astragalus membranaceus*; Lig, ligustrazine; I/R, ischemia/reperfusion; TTC, 2,3,5‐triphenyl tetrazolium chloride; MCAO/R, middle cerebral artery occlusion/reperfusion.

First, the effects of Ast+Lig treatment on the neurological deficit in cerebral I/R mice were evaluated. Ast (2 ml/kg) + Lig (1 ml/kg) was used based on previous studies (Cai et al., 2014; Pan et al., 2019). As shown in Figure 1a, the neurological deficit scores of the MCAO/R group were markedly higher than those of the Sham group (*p* < .05). The neurological deficits of mice in the Ast+Lig group or Mk‐801 (an inhibitor of NMDAR) group were significantly improved compared with the MCAO/R group (*p* < .05). Furthermore, TTC staining analysis showed that treatment with Ast+Lig or Mk‐801 significantly decreased infarct volumes (Figure 1b and c). Moreover, compared with the MCAO/R group, treatment with Ast+Lig, or Mk‐801 significantly decreased brain water content (Figure 1d). In addition, immunohistochemistry analysis showed that the morphology of the majority of neurons was clear and showed no morphological abnormalities. Conversely, most neurons in the MCAO/R group showed shrinkage, and the percentage of neurons was significantly decreased. Treatment with Ast+Lig or Mk‐801 significantly protected against changes in the morphology of neurons and prevented neuronal loss (Figure 1e and f).

### 3.2 Ast+Lig treatment suppresses the increase in NR2B expression and reduces neuronal death in the cerebral I/R hemisphere

**FIGURE 2:**
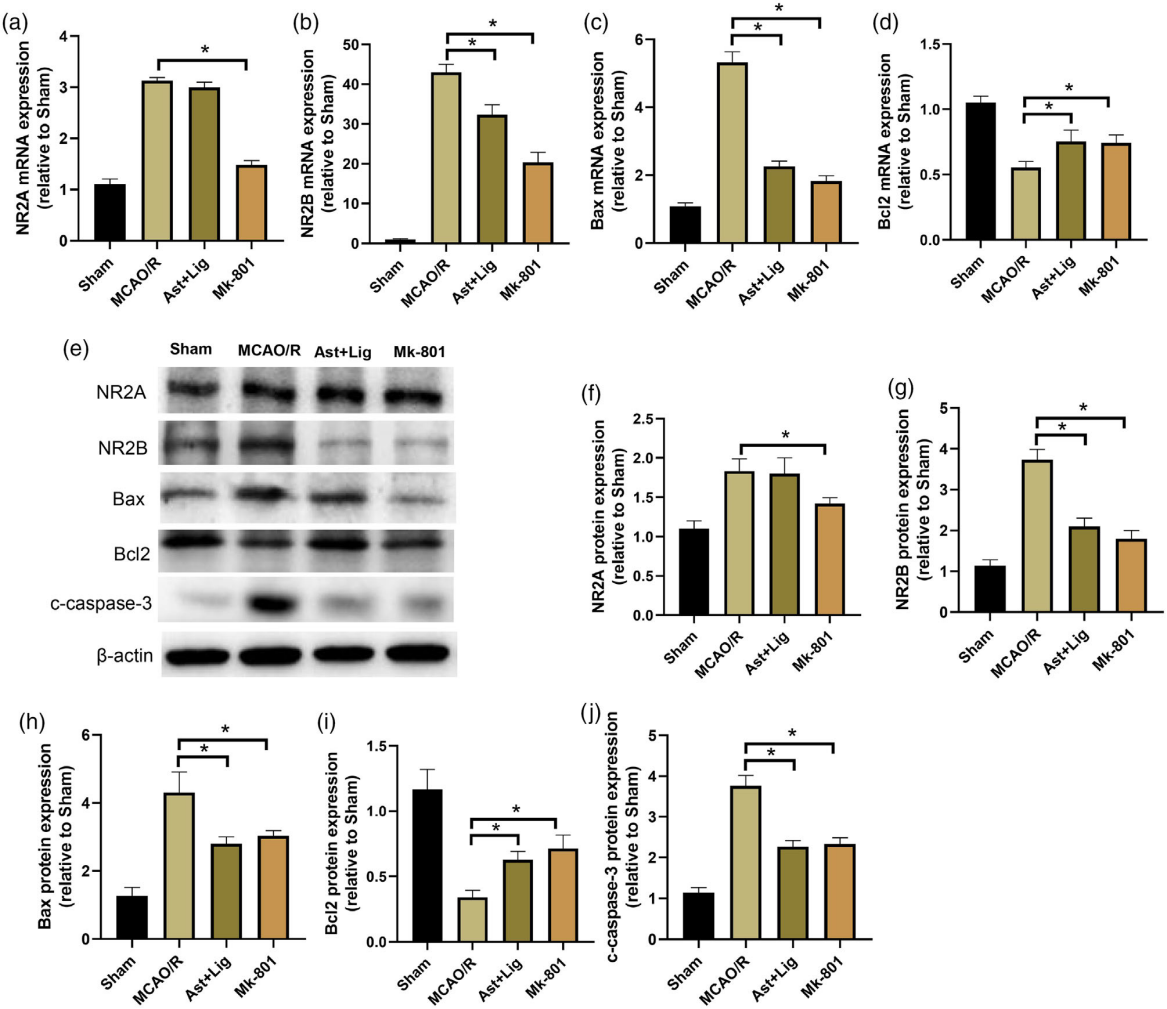
Ast+Lig treatment suppressed the increase in NR2B expression and neuronal death in the cerebral I/R hemisphere. Relative mRNA expression levels of (a) NR2A (*F* = 11.978), (b) NR2B (*F* = 232.770), (c) Bax (*F* = 277.575), and (d) Bcl‐2 (*F* = 31.558) were examined by RT‐qPCR. (e) Protein expression levels of NR2A, NR2B, Bax, Bcl‐2, c‐caspase‐3, and β‐actin were detected by western blotting. Relative expression levels of (f) NR2A (*F* = 18.312), (g) NR2B (*F* = 87.807), (h) Bax (*F* = 126.697), (i) Bcl‐2 (*F* = 34.548), and (j) c‐caspase‐3 (*F* = 11.978) were calculated and normalized to that of the respective β‐actin band. *n* = 3 per group. ^*^ *p* < .05 vs. MCAO/R group. Ast, *Astragalus membranaceus*; Lig, ligustrazine; I/R, ischemia/reperfusion; c‐caspase‐3, cleaved‐caspase 3; MCAO/R, middle cerebral artery occlusion/reperfusion.

Subsequently, we explored whether the protective effect of Ast+Lig on cerebral I/R injury was related to regulation of the NMDAR. As shown in Figure 2a–g, the mRNA and protein expression levels of NR2A and NR2B were both significantly increased in the MCAO/R group compared with those in the Sham group. Treatment with Ast+Lig suppressed the increase in NR2B expression, but not NR2A, at the mRNA and protein levels in the cerebral I/R hemisphere, consistent with the results of immunohistochemical analysis (Figure 2k and l). The upregulated NR2A and NR2B mRNA and protein expression levels were both significantly decreased in the Mk‐801 group (Figure 2a–g). Moreover, we also found that treatment with Ast+Lig or Mk‐801 significantly decreased the expression of proapoptotic proteins, Bax and c‐caspase‐3 expression, and increased the expression of the antiapoptotic protein, Bcl‐2, in the cerebral I/R hemisphere (Figure 2c–j). In addition, TUNEL staining showed that treatment with Ast+Lig suppressed cerebral I/R‐induced neuronal death (Figure 2k). The above results indicate that the protective effects of Ast+Lig on cerebral I/R injury may be related to the regulation of NR2B expression.

### 3.3 Ast+Lig treatment alleviates OGD/R‐induced neuronal injury

**FIGURE 3:**
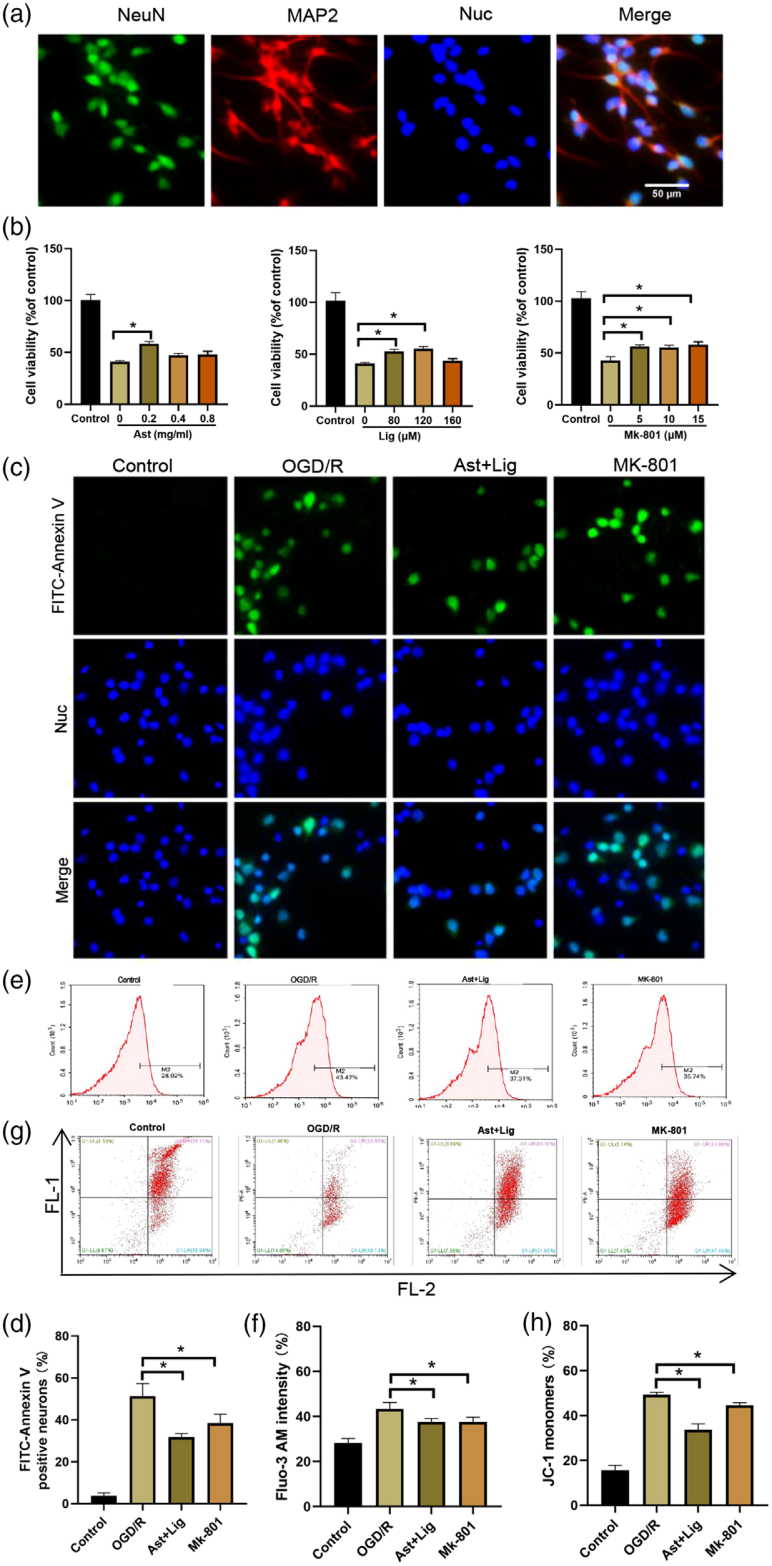
Ast+Lig treatment alleviated OGD/R‐induced neuronal injury. (a) Immunofluorescent staining of NeuN (green) and MAP2 (red) in the isolated rat cerebral cortical neurons. Nuclei were stained with DAPI (blue). (b) Neurons were treated with Ast (*F* = 24.714), Lig (*F* = 126.136), or Mk‐801 (*F* = 485.612) for 24 h following OGD/R. Cell viability was detected using a CCK‐8 assay. (c) Early apoptosis of neurons treated with Ast+Lig, or Mk‐801 for 24 h after OGD/R was detected using an immunofluorescent assay. Nuclei were stained with Hochest33342 (blue). (d) Quantification of FITC‐Annexin V positive cells displayed in (c) (*F* = 81.407). (e) The intracellular Ca^2+^ levels in neurons treated with Ast+Lig or Mk‐801 for 24 h following OGD/R were evaluated using flow cytometry with the fluorescent indicator Fluo‐3 AM. (f) Quantification of Fluo‐3 AM intensity in (e) (*F* = 25.292). (g) Mitochondrial membrane potential of neurons treated with Ast+Lig or Mk‐801 for 24 h following OGD/R was evaluated using flow cytometry with the fluorescent indicator JC‐1 to measure the degree in change in the ratio of red (FL‐2) to green (FL‐1) fluorescence. (h) Quantification of JC‐1 monomer intensity in (g) (*F* = 185.679). *n* = 3 per group. ^*^ *p* < .05 vs. OGD/R group. Ast, *Astragalus membranaceus*; Lig, ligustrazine; OGD/R, oxygen glucose deprivation/reperfusion.

The protective effects of Ast+Lig were further verified in primary rat cerebral cortical neurons following OGD/R to mimic cerebral I/R injury in vitro. The purity of isolated rat cerebral cortical neurons was confirmed by immunostaining with anti‐NeuN (neuronal marker) or Map2 (neuronal marker) antibodies (Figure 3a). Next, a CCK‐8 assay was used to determine the optimal doses of drugs. The results showed that treatment with different concentrations of Ast (0.2, 0.4, or 0.8 mg/ml), Lig (80 or 120 μM), or Mk‐801 (5, 10, or 15 μM) alone all significantly improved the cell viability of OGD/R‐injured neurons (Figure 3b). Thus, 0.2 mg/ml Ast + 120 μM Lig, and 5 μM Mk‐801 were used for the following in vitro experiments. Furthermore, the protective effects of Ast+Lig on neuronal injury were determined by staining with FITC‐Annexin V, which specifically recognized the apoptotic cells during the early stages of apoptosis (Zang et al., 2021), and the results showed that treatment with Ast+Lig or Mk‐801 significantly reduced OGD/R‐induced neuronal apoptosis (Figure 3c and d). Moreover, treatment with Ast+Lig or Mk‐801 suppressed the OGD/R‐induced Ca^2+^ influx (Figure 3e and f) and decreased the mitochondrial membrane potential (Figure 3g and h). The above results indicate that the combination of Ast+Lig treatment alleviated OGD/R‐induced neuronal injury.

### 3.4 Ast+Lig treatment inhibits the upregulation of NR2B and proapoptotic proteins in neurons following OGD/R injury

**FIGURE 4:**
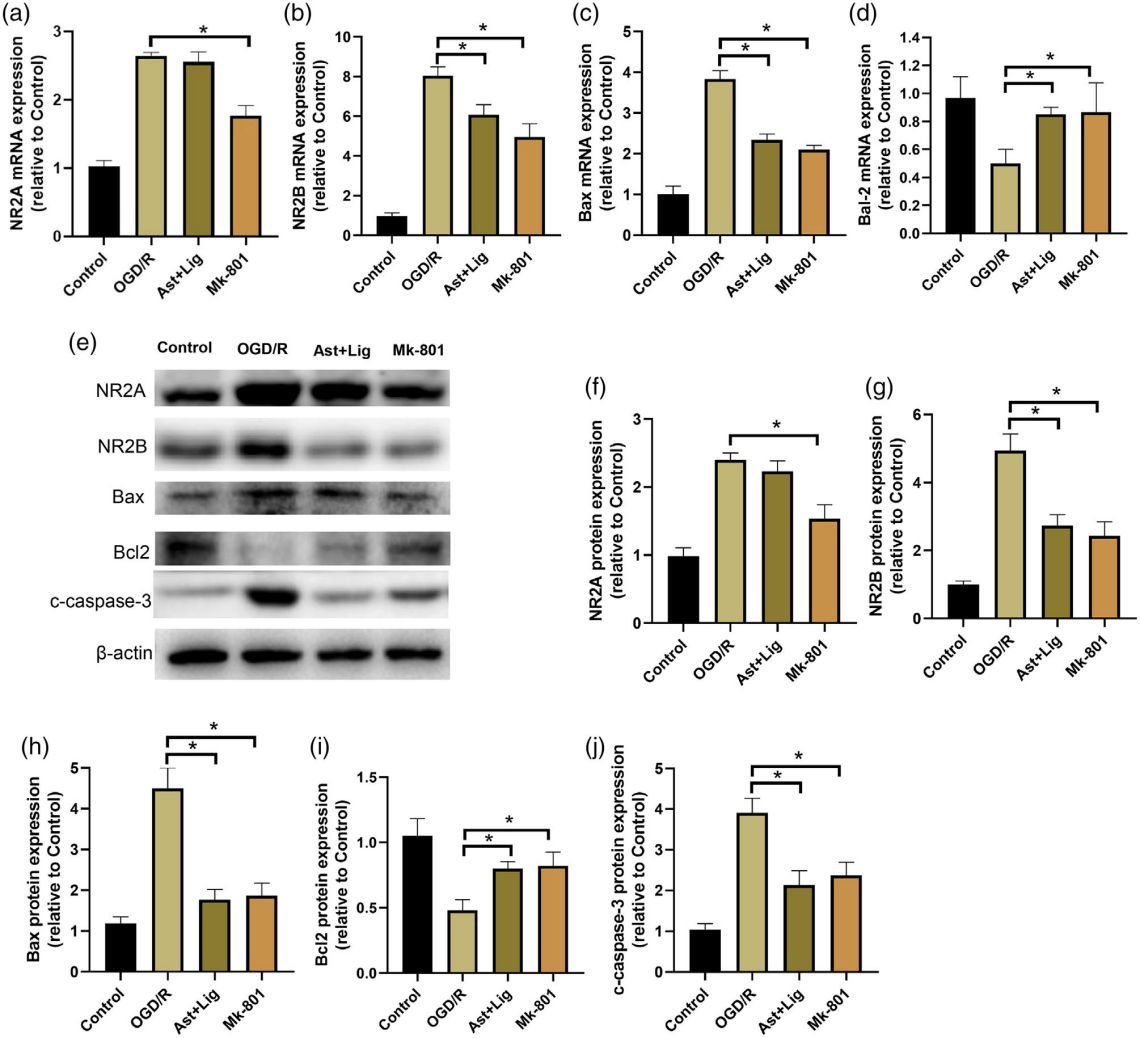
Ast+Lig treatment inhibited the upregulation of NR2B and proapoptotic protein in neurons with OGD/R injury. Relative mRNA expression levels of (a) NR2A (*F* = 123.687), (b) NR2B (*F* = 115.938), (c) Bax (*F* = 139.924), and (d) Bcl‐2 (*F* = 6.298) in the neurons treated with Ast+Lig, or Mk‐801 for 24 h following OGD/R. (e) Protein expression levels of NR2A, NR2B, Bax, Bcl‐2, c‐caspase‐3, and β‐actin were detected by western blotting. Relative expression levels of (f) NR2A (*F* = 55.577), (g) NR2B (*F* = 59.803), (h) Bax (*F* = 60.463), (i) Bcl‐2 (*F* = 17.380), and (j) c‐caspase‐3 (*F* = 44.032) were calculated by normalizing expression to that of the respective β‐actin band. *n* = 3 per group. ^*^ *p* < .05 vs. OGD/R group. Ast, *Astragalus membranaceus*; Lig, ligustrazine; OGD/R, oxygen glucose deprivation/reperfusion.

The effects of Ast+Lig treatment on NMDAR expression in neurons following OGD/R were investigated. Ast+Lig notably suppressed the increase in NR2B mRNA and protein expression levels in neurons treated with OGD/R (Figure 4b, e, and g), consistent with the results of the in vivo analysis (Figure 2b, e, and g). In addition, treatment with Ast+Lig or Mk‐801 also significantly decreased the expression of the proapoptotic proteins, Bax and c‐caspase‐3, and increased the expression of the antiapoptotic protein, Bcl‐2, in neurons following OGD/R (Figure 4c–j). Overall, these results indicated that Ast+Lig treatment inhibited the upregulation in NR2B and proapoptotic protein expression in neurons following OGD/R injury.

### 3.5 Ast+Lig treatment alleviates the reduction in ERK and CREB phosphorylation in the cerebral I/R hemisphere

**FIGURE 5:**
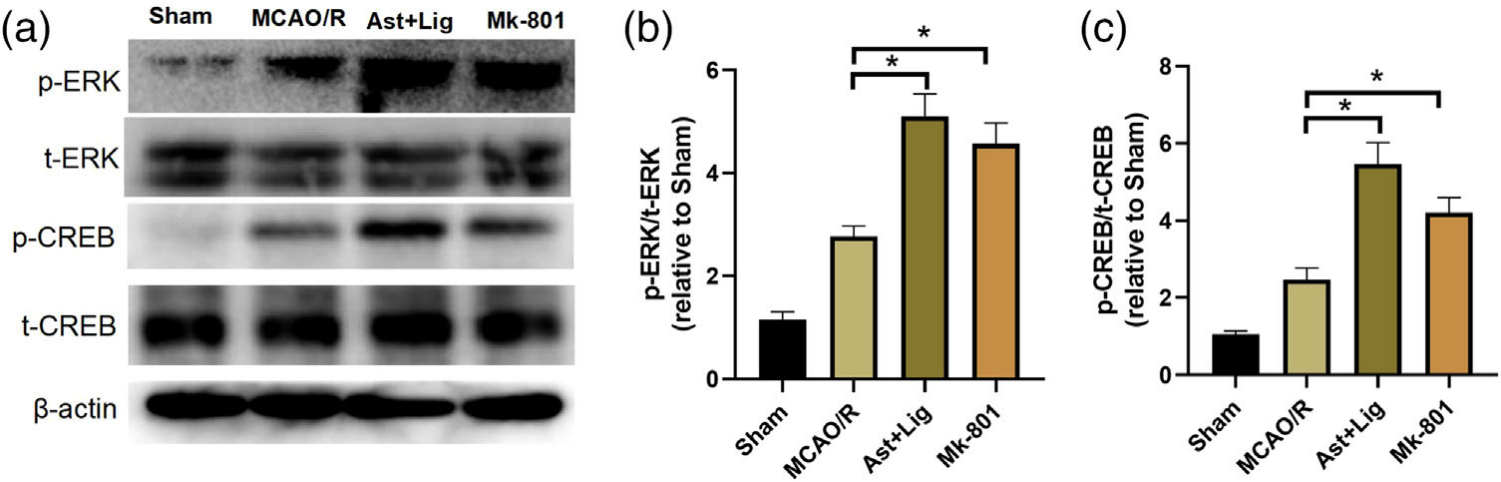
Ast+Lig treatment alleviated the reduction in ERK and CREB phosphorylation in the cerebral I/R hemisphere. (a) Protein expression levels of t‐ERK, p‐ERK, t‐CREB, p‐CREB, and β‐actin in the peri‐infarcted area. (b) Relative expression levels of p‐ERK was calculated by normalizing to the respective t‐ERK band (*F* = 92.554). (c) Relative expression levels of p‐CREB were calculated by normalizing to that of the respective t‐CREB band (*F* = 79.592). *n* = 3 per group. ^*^ *p* < .05 vs. MCAO/R group. Ast, *Astragalus membranaceus*; Lig, ligustrazine; MCAO/R, middle cerebral artery occlusion/reperfusion; t‐, total.

Given that ERK/CREB signaling plays an essential role in NMDAR‐dependent neuronal survival (Li et al., 2014; Liu et al., 2017; Wu et al., 2020), the effects of Ast+Lig treatment on ERK/CREB signaling in vivo were further explored. As shown in Figure 5a–c, compared with the Sham group, the protein expression levels of p‐ERK and p‐CREB were significantly increased in the brain tissues following cerebral I/R, and treatment with Ast+Lig or Mk‐801 further increased the expression of these proteins.

### 3.6 Ast+Lig treatment alleviates the reduction in ERK and CREB phosphorylation in neurons following OGD/R injury

**FIGURE 6:**
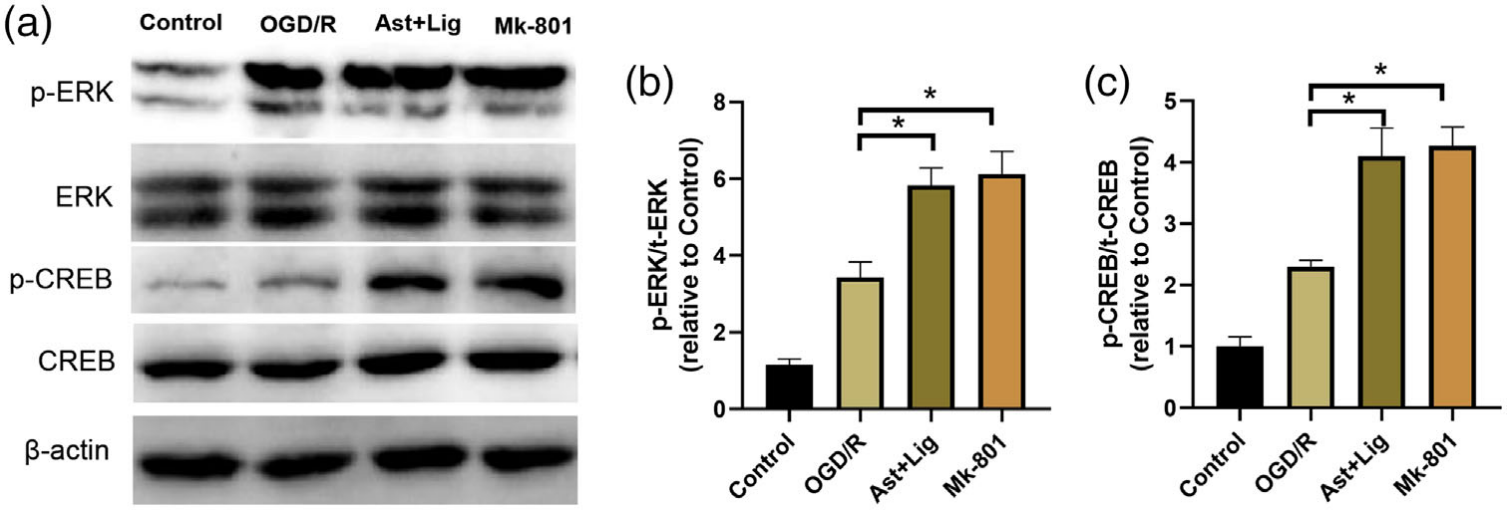
Ast+Lig treatment alleviated the reduction of ERK and CREB phosphorylation in neurons following OGD/R injury. (a) Protein expression levels of t‐ERK, p‐ERK, t‐CREB, p‐CREB, and β‐actin in the neurons treated with Ast+Lig, or Mk‐801 for 24 h following OGD/R injury were detected by western blotting. (b) Relative expression levels of p‐ERK were calculated by normalizing to that of the respective t‐ERK band (*F* = 88.789). (c) Relative expression levels of p‐CREB were calculated by normalizing to that of the respective t‐CREB band (*F* = 86.357). *n* = 3 per group. ^*^ *p* < .05 vs. OGD/R group. Ast, *Astragalus membranaceus*; Lig, ligustrazine; OGD/R, oxygen glucose deprivation/reperfusion; t‐, total.

Finally, the effects of Ast+Lig treatment on ERK/CREB signaling in vitro were examined. As shown in Figure 6a–c, compared with the Control group, the protein expression levels of p‐ERK and p‐CREB were significantly increased in the neurons following OGD/R, and treatment with Ast+Lig or Mk‐801 further increased the expression of these proteins. Overall, these results indicate that Ast+Lig treatment mitigates cerebral ischemia‐reperfusion injury via regulation of NR2B‐ERK/CREB signaling.

## 4 DISCUSSION

The present study showed that Ast+Lig treatment significantly ameliorated neurological deficits, decreased infarct volumes, suppressed neuronal damage and Ca^2+^ influx, and maintained the mitochondrial membrane potential in vivo and in vitro following cerebral I/R injury. Furthermore, treatment with Ast+Lig prevented the increase in NR2B expression and promoted the phosphorylation of ERK and CREB, as well as increasing their mRNA expression levels in vivo and in vitro following cerebral I/R injury. This study demonstrated that Ast+Lig treatment mitigated cerebral I/R injury via regulation of NR2B‐ERK/CREB signaling.

Treatment with rt‐PA for acute ischemic stroke in the clinical setting generally results in blood reperfusion to the brain, which triggers secondary damage to the brain and causes more serious brain dysfunction and nerve defects (An et al., 2016). Thus, it is necessary to explore effective therapies to mitigate these complications. Buyang Huanwu decoctions are widely used in Chinese medicinal decoctions for the treatment of cerebral ischemia (Dou et al., 2018). Ast+Lig, essential constituents of Buyang Huanwu decoctions, have been found to possess protective effects against neurological dysfunction, such as relieving the damage of the Blood‐Brain Barrier in rats (Qu et al., 2009) and decreasing cerebral edema post‐I/R (Li et al., 2013). In addition, it has been shown that Ast can reduce the volume of cerebral infarction by inhibiting neuronal apoptosis in the ischemic area (Liu et al., 2013). Lig can dilate blood vessels, prevent platelet aggregation, and act as an antithrombotic (Li et al., 2019). Our recent studies found that the combination of Ast+Lig inhibits thrombolysis‐induced hemorrhagic transformation via PKCδ/Marcks signaling and suppresses the inflammatory response in cerebral ischemia rats (Cai et al., 2014; Pan et al., 2020). Here, it was shown that Ast+Lig treatment mitigated neuronal damage in cerebral I/R injury via regulation of NR2B‐ERK/CREB signaling. Given the complex pathogenesis of cerebral I/R injuries, such as excessive apoptosis, oxidative stress, excitotoxicity, acidosis, and inflammation, and the several protective effects of Ast+Lig on neurological dysfunction, it is suggested that Ast+Lig systemically modulates multiple targets to relieve cerebral I/R injury. Our study further confirmed the significance of Buyang Huanwu decoctions (and potentially other Chinese medicinal decoctions) for the management of stroke. However, the underlying molecular mechanism of Buyang Huanwu decoctions still requires deeper investigation.

NMDARs are cation channels that are gated by the neurotransmitter glutamate and they play essential roles in regulating neuronal development, synaptic transmission, and plasticity (Wang et al., 2015). During cerebral I/R injury, NMDARs are overactivated by excessive glutamate release, resulting in Ca^2+^ overload in neurons, mitochondrial dysfunction, and eventually neuron death (Wroge et al., 2012). Previous studies found that the NMDAR‐mediated ERK1/2‐CREB signaling pathway is the key to controlling the antiapoptotic Bcl‐2 protein family in blocking cell apoptosis (Ninomiya et al., 2010), and the survival rate of neurons in the hippocampal CA1 region of rodents with cerebral ischemia is positively associated with the levels of p‐ERK and pCREB (Zhan et al., 2013). Resveratrol and Cytisine have been found to relieve cerebral I/R‐induced damage via the ERK‐CREB signaling pathway (Li et al., 2016; Zhao et al., 2018). In the present study, it was shown that Ast+Lig could specifically inhibit the upregulation of NR2B expression induced by cerebral I/R, but not NR2A, and it further enhanced the levels of p‐ERK and pCREB to alleviate brain damage. Previously, studies reported that the Src‐family of nonreceptor protein‐tyrosine kinases regulated the tyrosine phosphorylation of the NR2A and NR2B during cerebral I/R injury, thereby controlling the upregulation of receptor and channel function (Ali & Salter, 2001; Yu et al., 1997). Whether Ast+Lig treatment‐mediated regulation of NR2B expression is related to the Src‐family of nonreceptor protein‐tyrosine kinases, and the detailed molecular mechanism underlying this association should be explored in future studies.

The present study has some limitations. First, we only preliminarily discussed the interventional effect of Ast+Lig in a small sample size, thus limiting the statistical power of the experiments, thus a larger sample size should be used in future studies. Second, two different species were used for the in vivo and in vitro studies. We selected rat primary neurons for experimentation in vitro due to technical limitations, but in future studies mouse primary neurons will be used. Third, as a limitation of the experimental design, Ast or Lig alone groups were not included to compare their effects alone with the combined effect of both as described previously (Cai et al., 2014). In addition, how blocking NR2B or ERK/CREB signaling affected the protective role of Ast+Lig in I/R injury remains to be determined.

In conclusion, the results of the present study suggest that Ast+Lig treatment mitigated cerebral I/R‐induced neuroexcitatory toxicity and promoted neuronal survival. The mechanism of this effect was, at least in part, related to the regulation of NR2B‐ERK/CREB signaling. Although a detailed molecular mechanism has been previously described for the protective effects of Ast+Lig on cerebral I/R injury, these findings highlight the efficacy and significance of the Buyang Huanwu decoction in the treatment of stroke.

## AUTHOR CONTRIBUTIONS

XT, SX, HW, and YL performed the experiments and wrote the manuscript, analyzed the data, prepared the figures, and interpreted the data. ZL, SS, and RP participated in performing the experiments and analyzing the data. YH conceived and designed the study and revised the manuscript. JC designed the study, provided technical support, and was involved in writing and revising the manuscript.

## COMPETING INTERESTS

The authors declare that they have no competing interests.

### PEER REVIEW

The peer review history for this article is available at https://publons.com/publon/10.1002/brb3.2867.

## Data Availability

The data sets used and/or analyzed during the present study are available from the corresponding author on reasonable request.
